# Contribution and Mobilization of Mesenchymal Stem Cells in a mouse model of carbon tetrachloride-induced liver fibrosis

**DOI:** 10.1038/srep17762

**Published:** 2015-12-08

**Authors:** Yan Liu, Xue Yang, Yingying Jing, Shanshan Zhang, Chen Zong, Jinghua Jiang, Kai Sun, Rong Li, Lu Gao, Xue Zhao, Dong Wu, Yufang Shi, Zhipeng Han, Lixin Wei

**Affiliations:** 1 Tumor Immunology and Gene Therapy Center, Eastern Hepatobiliary Surgery Hospital, Second Military Medical University, Shanghai, 200438, PR China; 2 Central laboratory, Ren Ji Hospital, School of Medicine, Shanghai Jiao Tong University, Shanghai, China; 3 Department of Hepatic Surgery, Eastern Hepatobiliary Surgery Hospital, The Second Military Medical University, 200438, Shanghai, China; 4 Institute of Health Sciences and Shanghai Institute of Immunology, Chinese Academy of Sciences and Shanghai Jiao Tong University School of Medicine, 225 South Chongqing Road, Shanghai 200225, China

## Abstract

Hepatic fibrosis is associated with bone marrow derived mesenchymal stem cells (BM-MSCs). In this study, we aimed to determine what role MSCs play in the process and how they mobilize from bone marrow (BM). We employed a mouse model of carbon tetrachloride(CCl_4_)-induced liver fibrosis. Frozen section was used to detect MSCs recruited to mice and human fibrotic liver. Alanine aminotransferase (ALT) and aspartate aminotransferase (AST) was detected to assess liver function. It was found that MSCs of both exogenous and endogenous origin could aggravate liver fibrosis and attenuate liver damage as indicated by lower serum ALT and AST levels. Stromal cell–derived factor-1 (SDF-1α)/ CXCR4 was the most important chemotactic axis regulating MSCs migration from BM to fibrotic liver. Frozen section results showed that the migration did not start from the beginning of liver injury but occured when the expression balance of SDF-1α between liver and BM was disrupted, where SDF-1α expression in liver was higher than that in BM. Our findings provide further evidence to show the role of BM-MSCs in liver fibrosis and to elucidate the mechanism underlying MSCs mobilization in our early liver fibrosis mice model induced by CCl_4_.

Liver fibrosis is the consequence of a sustained wound-healing response to chronic liver injury. Progressive liver fibrosis leads to cirrhosis and hepatocellular carcinoma^1^. Currently, several antifibrotic drugs are in development for the treatment of liver fibrosis but the efficacy has not been proven in patients^2^. Further understanding of the cellular and molecular mechanism of liver fibrosis may lead to the development of more effective treatment.

There is accumulating evidence suggesting that liver fibrogenesis engages a range of cell types and mediators to encapsulate injury. These key cells involved in fibrogenesis include hepatic stellate cells (HSCs), myofibroblasts, kuffer cells and MSCs^3^,^4^,^5^,^6^. MSCs are believed to be important cells associated with liver fibrogenesis^7^. However, so far the role of MSCs in liver fibrosis is still controversial. It is reported that MSCs could promote the development of liver fibrosis^6^,^8^, while others suggest the contrary^9^,^10^. Besides, it is also observed that MSCs have no influence in the fibrogenesis^11^,^12^. For review on MSCs and fibrosis see Usunier *et al.*^13^.

In addition, the mechanism of MSCs mobilization during fibrogenesis remains incompletely defined. We know that MSCs mainly existing in bone marrow have the capacity of pluripotent differentiation, which can differentiate into adipocytic, chondrocytic, and osteogenic lineages and potentially other lineages including epithelial, myofibroblast, and neuronal lineages^8^,^14^. In response to disease or tissue injury, these cells are mobilized from the bone marrow and recruited into tissues where they contribute either to tissue repair or disease progression^15^,^16^. Main mechanisms of protection of liver injury are antioxidative process, vasculature protection, hepatocyte differentiation, and trophic effects.^17^ Liver fibrosis is a chronic disease and has long duration. During the process, MSCs continuously trend to injured liver, which necessarily requires quantities of MSCs. Therefore, cell proliferation is one of the important processes of MSCs mobilization. Studies suggested that some angiogenesis-related cytokines might be related to MSCs proliferation. Moreover, we know that injured liver culminates in angiogenesis and vascular reorganization, and kinds of cytokines related to angiogenesis are produced and secreted extracelluarly^18^. So we hypothesized that some angiogenesis-related cytokine produced in liver injury contributed to MSCs proliferation in BM.

Mobilization of MSCs is a multistage process following MSCs proliferation, the release of MSCs from BM is another key matter. It is reported that some chemokines and their receptors were relevant to MSCs migration^19^,^20^,^21^. However, it is still unknown which chemokine axis is the critical one contributing to MSCs migration from BM and their recruitment to fibrotic liver.

To define these questions, we used several kinds of animal models, including CCl_4_ induced liver fibrosis, bone marrow transplantation and the model of induced endogenous MSCs in mice. The aims of these models and related detection *in vitro* were to investigate the mechanism underlying MSCs mobilization and its role in early liver fibrosis.

## Materials and Methods

### Mice

Wild type and EGFP-transgenic male BALB/c mice (20–25 g, 8 to 10-week) were purchased from Shanghai Experimental Animal Center of the Chinese Academy of Sciences. The mice were fed on a standard diet and acclimated in a quiet quarantine room for 1 week before the experiments. The committee for animal research also approved the experiments for our study. Animal experimentation methods were carried out in accordance with the approved guidelines. There were no ethic issues during our experiments.

### Reagents

The PE-conjugated anti-human anti-SSEA-4 antibody, PE-conjugated anti-human anti-CD105, -CD34 antibodies as well as FITC-conjugated anti-human anti-CD45, -CD90 antibodies were purchased from eBioscience (San Diego, CA, USA), Avastin from Roche (Basel, Switzerland), vascular endothelial growth factor (VEGF) antibody from Biolegend (San Diego, CA, USA), AMD3100 from Sigma(St. Louis, MO, USA) and VEGF, SDF-1 from Peprotech (Rocky Hill, NJ, USA).

### Cells

MSCs of WT and EGFP-tansgenic BALB/c mice were generated from bone marrow of tibia and femur of 6–10-week-old mice. To obtain MSCs clones, we then picked and expanded these cells as previously reported^22^.

The ability of MSCs to differentiate into osteoblasts^23^ and adipocytes^24^ was confirmed prior to use. The differentiated osteoblasts were stained with von kossa, the adipocytes with Oil Red according to published protocols^25^. As analyzed by flow cytometry (FACS), the mice MSCs surface antigen profile was consistently CD34-, CD45-, CD29+, CD90+, CD105+.

### Animal models

Bone marrow transplantation (BMT) model: WT-mice aged 10 weeks received lethal irradiation (8 Gray, 30 min), and immediately received transplantation of enriched 2 × 10^6^ BMSCs obtained from 8-week-old EGFP mice and 1 × 10^7^ whole BM cells from WT mice by a tail vein injection. These mice were used to further researches in one or two months after BMT.

Induced liver fibrosis model: Mice received intragastrical administration (i.g.) of 5 uL CCl_4_/olive oil mixture (1:4 v/v) per gram body weight twice per week for indicated time period.

MSCs administration models: (1)Exogenous administration model: mice were divided into four groups, ctrl group, ctrl+MSCs group, CCl_4_ group and CCl_4_+MSCs group. 1 × 10^5^ MSCs were injected by tail vein once every two weeks at the third week after CCl_4_ administration. Mice were sacrificed at the end of the fourth week. (2)Endogenous induction model: at the fourth week after BMT, mice were divided into two groups, ctrl group and CCl_4_ group. CCl_4_ was administered to mice for four weeks.

VEGF and AMD3100 administration model: mice were administered with oil and CCl_4_ for four weeks separately. The oil and CCl_4_ consumption mice were both divided into three groups: ctrl group, VEGF and AMD3100 group, Avastin and AMD3100 group. VEGF (2.5 μg/mouse, i.v.) was injected for 4 consecutive days at the first and third week, AMD3100 (5 mg/kg i.p) was injected twenty-four hours after the last VEGF injection^26^.

To confirm the time of MSCs migration, mice fibrosis model was induced for five weeks as described in the passage 2. Mice were killed at the end of 1, 2, 3, 4, 5 week during CCl_4_ administration. Three days before killed, mice were given 1 × 10^5^ MSCs from EGFP-mice (i.v.) and frozen sections of livers and some other solid organs were made.

For all the animal models, 6mice were used for every group.

### Flow Cytometry Analysis

The fresh specimens of human hepatic cirrhosis and normal liver tissues were transferred to a petri dish, where the tissue was gently minced and filtered (100 mm) to remove large aggregates, the cell suspension was filtered (40 mm) and nonparenchymal cells were separated by discontinuous density gradients of Percoll (Pharmacia Biotech). The SSEA-4 antibody was added to the final cell suspension at 0.1 μg/10^6^ cells and incubated at 4 °C for 30 minutes before washing with blocking buffer, and then stained cells were analyzed on a FACS Aria (Becton Dickinson, San Jose, CA). In the sorting experiments, cells were purified based on the expression of SSEA-4 (positives and negatives). For clonal analysis, SSEA-4^+^ cells were deposited into single wells of a 96-well dish. Wells with single cell colonies were harvested and expanded into clonal cell lines. CD105, CD34, CD45 and CD90 were applied for characterization of human MSCs.

The WT-BALB/c mice, which have been transplanted with EGFP-MSCs, were induced hepatic fibrosis by received CCl_4_. The fresh specimens of mouse hepatic cirrhosis and normal liver tissues were digested with collagenase to produce cell suspension, which was used to identify GFP-positive MSCs by FACS^27^.

Bone marrow and blood cells of the GFP-chimetic mice were obtained from mice with and without CCl_4_ treatment. These cells were examined by FACS to analysis the percentage of GFP positive cells after the erythrocytes were removed by erythrocyte lysis buffer.

### Immunofluorescence and Immunohistochemistry

Immunofluorescent staining for SSEA-4 on human mesenchymal stem cells (HMSCs) cell lines and in human cirrhosis tissue was performed as previously described^28^. Immunohistochemistry staining for SSEA-4, VEGF, SDF-1α was performed as previously described by Barraud *et al.*^29^.

### Sirius Red Staining

Liver tissues were fixed in 10% buffered formalin, embedded in paraffin, and sectioned at 5μm thickness. Sections were stained with Sirius red solution (0.1% Direct Red 80 in saturated picric acid) to visualize collagen deposition.

### Measurement of Hepatic Hydroxyproline Content

The hepatic hydroxyproline level was determined by using the hydroxyproline detection kit (Nanjing Jiancheng Bioengineering Institute, Nanjing, China). The methods were carried out in “accordance” with the approved guidelines

### Wound Healing and Transwell Assay

The methods for wound healing and the Transwell assay have been described^28^. These experiments were performed in triplicate.

### Real-time PCR Analyses

The cells were collected to extract the total cellular RNA with Trizol Reagent (Invitrogen, Carlsbad, CA, USA). cDNA was synthesized using MMLV reverse transcriptase(Promega, WI, USA), 2 μg total RNA and oligo dT18-primers. Real-time PCR was performed in triplicate using the SYBR PrimeScript RT–PCR Kit (Takara, Dalian, China). Two-microliter aliquots of cDNA were used and the primers for VEGF were as follows: forward primer 5′-TAC TGC TGT ACC TCC ACC TCC ACC ATG-3′ and reverse primer 5′-TCA CTT CAT GGG ACT TCT GCT CT-3′, and all other primers were listed in supplementary table 1 and 2. Total sample RNA was normalized to endogenous β-actin mRNA. Thermocycler conditions included an initial hold at 50 °C for 2 minutes and then 95 °C for 10 minutes which was followed by 40 cycles of a two-step PCR program of 95 °C for 15 seconds and 60 °C for 60 seconds on an Mx4000 system (Stratagene, La Jolla, CA), on which data were collected and quantitatively analyzed. Expression level of mRNA was presented as fold change relative to an untreated control.

### Statistical analysis

Statistical analysis of the data was done by using GraphPad Prism 4. Student’s t-test was used to compare the mean values of two groups. Data between three or more groups were compared using the one-way analysis of variance, followed by the Dunnett’s post hoc test. Final values are expressed as mean ± s.d. A difference of at least P < 0.05 was considered statistically significant.

## Results

### MSCs Derived From BM are Present in Fibrotic Liver

**Figure 1:**
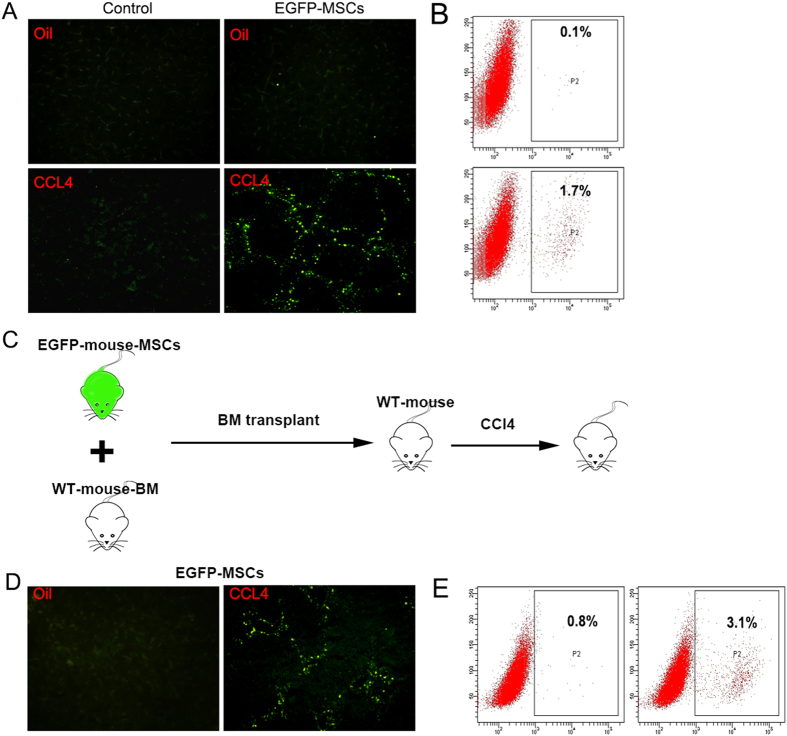
Both Exogenous and Endogenous BM-MSCs are Present In Fibrotic Mouse Liver. Hepatic fibrosis was induced in WT-BALB/c mice by administration of CCl_4_ for 4 weeks. Control group received olive oil treatment. Then EGFP-positive MSCs were infused via tail vein injection. (**A**,**B**)The fluorescence microscope and flow cytometry were employed to detect EGFP-positive MSCs in livers of each groups. (**C**) Mice model schematic. WT-BALB/c mice were lethally irradiated and received BMT including EGFP-positive BMSCs and EGFP-negative whole BM cells. Then hepatic fibrosis was induced by administration of CCl_4_ for 4 weeks. (**D**) EGFP-positive MSCs appear in the stroma of fibrosis livers.(The mouse drawing in C was drawn by Xue Yang).

To investigate the role of MSCs, we first detect the source of MSCs in liver fibrosis. In our study, mice BM-MSCs were identified based on spindle-shaped fibroblastic morphology and the capability of differentiating into osteoblasts and adipocytes (Figure S1A and S1B) and on the phenotypes(Figure S1C). At the end of the sixth week after inducing liver fibrosis with CCl_4_, 5 × 10^5^ exogenetic EGFP-MSCs were injected into the tail vein of the mice. Two days later, frozen section showed that large numbers of EGFP-positive cells located in fibrotic liver but were not found in normal mice liver (Fig. 1A) and the tissues of heart, brain, kidney and lung of CCl_4_-treated mice (data not shown). Flow cytometry detected the quantity of EGFP-cells in normal (control) and fibrotic livers (Fig. 1B). The data suggested that MSCs could be recruited to fibrotic liver. As shown in Fig. 1C, following lethal irradiation, wild type-mice (WT-mice) received whole BM transplants (BMT) from WT-mice and EGFP-MSCs from donor EGFP-mice at the age of 8 weeks. After 4 week, mice started to receive CCl_4_ administration to induce fibrosis. After six weeks, frozen section showed that significant numbers of EGFP-positive cells located in fibrotic mouse liver(Fig. 1D). These results suggest that can recruit endogenous and exogenous MSCs can be recruited to liver during liver fibrogenesis induced by CCl_4_. Furthermore, MSCs in human fibrotic tissues was investigated(Figure S3).

### Exogenetic MSCs Aggravate the Degree of Early Liver Fibrosis and Decrease Liver Injury in Mice

**Figure 2:**
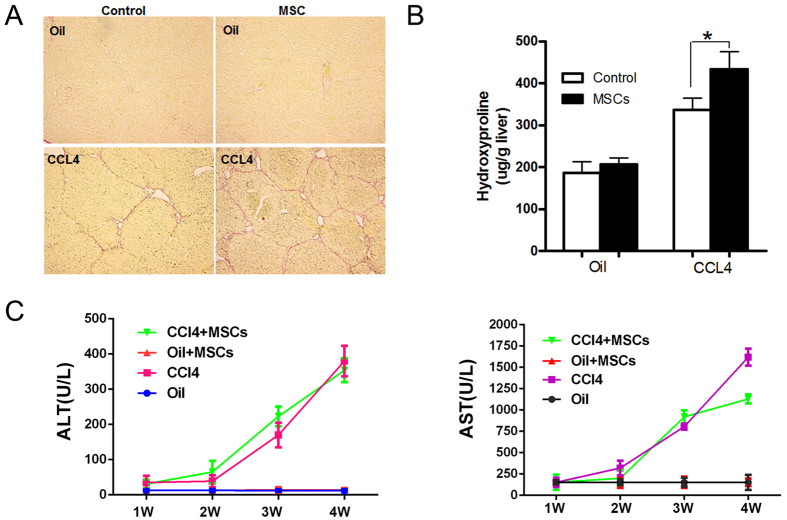
Exogenous MSCs Aggravate Hepatic Fibrosis and Decrease Liver Injury in Mice. (**A**) The significantly increased amount of ECM was confirmed by Sirius red staining (×100) after MSCs delivery. Data shown are the representative of 6 animals. (**B**) The amount of liver hydroxyproline was detected in MSCs injected group compared with that of control groups. (**C**) Serum levels of AST and ALT were determined to indicate the extent of liver damage caused by CCl_4_ administration and the role of MSCs in attenuating the damage (*P < 0.05).

The role of MSCs in liver fibrosis is still controversial. In order to explore this, we injected MSCs to mice once every two weeks during the six weeks of CCl_4_-induced fibrogenesis. At the end of the sixth week, we assessed the degree of fibrosis in liver tissue by sirius red staining. The result showed that exogenetic MSCs could aggravate mice liver fibrosis in this model (Fig. 2A,B).

We found that liver damage of mice administrated with MSCs was less than that of control as indicated by lower AST and ALT levels (Fig. 2C). Therefore, we may infer BM-MSCs that home to injured liver result in aggravation of fibrosis and may serve as a protection from hepatic damage by CCl_4_.

### SDF-1α/ CXCR4 Is the Key Chemotactic Axis Regulating MSCs Migration from BM to Liver

**Figure 3:**
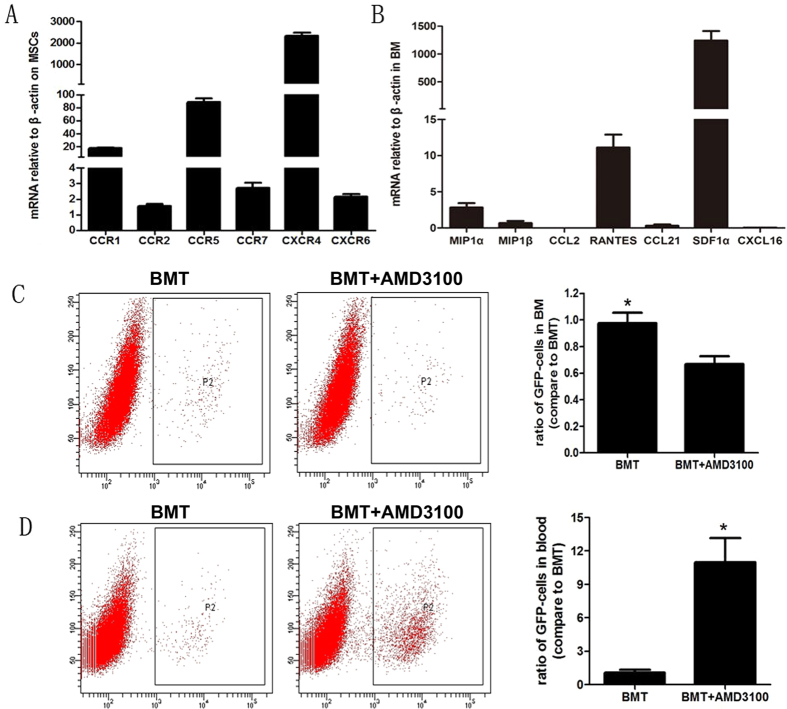
High SDF-1α expression in BM retains MSCs in BM. The expressions of some key chemokine receptors on MSCs (**A**) and their ligands in BM (**B**) were detected by Real-time PCR. 30 minutes after the CXCR4 antagonist-AMD3100 was administrated in BMT-mice, the percentages of EGFP-MSCs in BM (**C**) and in blood (**D**) were determined by flow cytometry. (**C**,**D**) A representative photograph is shown in the left panel. Quantification of the experiments is shown in the right panel (*P < 0.05).

**Figure 4:**
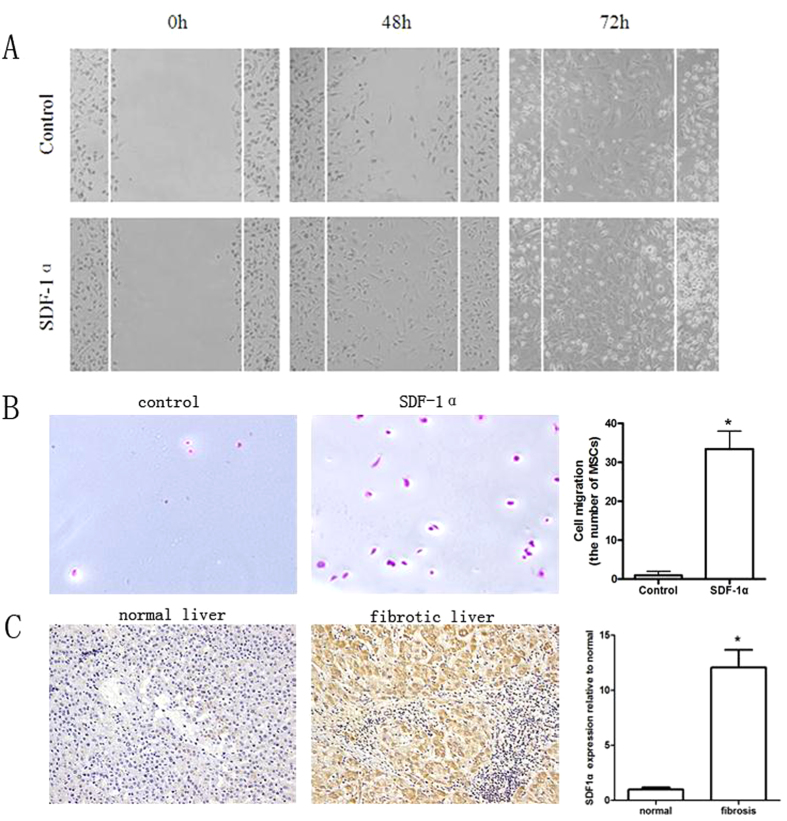
SDF-1α shows strong chemotaxis for MSCs *in vitro.* (**A**) The wound healing assay was employed to determine the migration of MSCs. MSCs were monitored at the 0th, 48th and 72th hour, which were co-cultured with or without SDF-1α to determine the rate of migration into the scratched area. (**B**) The effect of SDF-1α on invasiveness of MSCs was determined using Transwell assay. A representative photograph is shown in the left panel. Quantification of three independent experiments is shown in the right panel. (×200 magnification). (**C**) IHC was employed to examine SDF-1α expression in hepatic cirrhosis tissue from mice (*P < 0.05).

It is reported that MSCs migration is closely related to some chemotactic cytokine^19^,^20^,^21^. So we designed 16 primers (Supplementary Table 1) in CCR and CXCR families to screen the important receptors expressed on MSCs by Real-time PCR. The result showed that only 6 receptors were expressed on MSCs, and the expression of CXCR4 being the highest (Fig. 3A). Then we detected the expression of the ligands related to the 6 receptors in BM (Supplementary Table 2). We found that the expression of SDF-1α, the ligand of CXCR4, was significantly higher than other ligands (Fig. 3B). So we speculated that SDF-1α/CXCR4 axis was the critical reason why numerous MSCs stayed in bone marrow. To verify the speculation, the number of EGFP-MSCs in BM and peripheral blood was determined 30 minutes after administration of CXCR4 antagonist AMD3100 in BMT-mice. Compared with control, AMD3100 administration significantly decreased the number of EGFP-MSCs in BM (Fig. 3C), while the circulating number of MSCs in mice with AMD3100 administration was increased more than 10 folds (Fig. 3D). These data suggest that MSCs migration can be regulated by disrupting the SDF-1α/ CXCR4 axis. Furthermore, we performed wound healing assay and Transwell assay to provide *in vitro* evidence that SDF-1α could regulate MSCs migration. The data showed that SDF-1α treatment could accelerate the wound healing process (Fig. 4A) and stimulate the migration of MSCs to the lower chamber (Fig. 4B).

**Figure 5:**
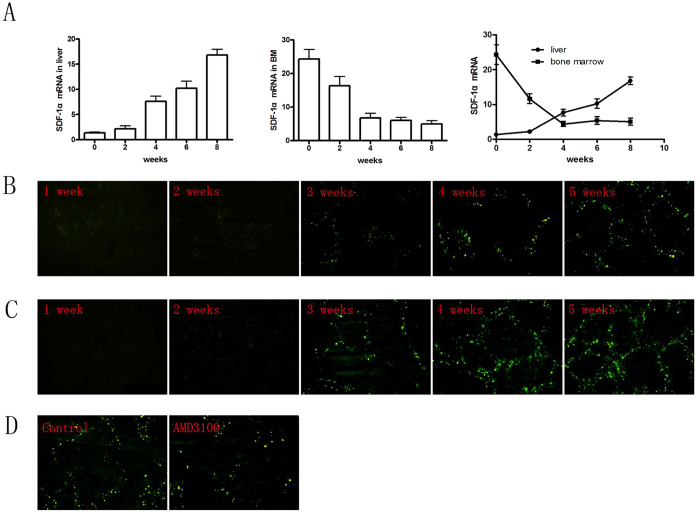
Dynamic changes of SDF-1α with development of hepatic fibrosis. (**A**) Real-time PCR was used to detect dynamic changes of SDF-1α in liver and bone marrow with development of hepatic fibrosis at the 0th, 2nd, 4th, 6th and 8th week after CCl4 administration. The mRNA expression was normalized against β-actin. (**B**) At different time points during CCl4-induced liver fibrosis in BMT mice, mice were sacrificed and frozen section of the liver tissue was made, and then the homing of EGFP-MSCs were observed under fluorescence microscope. (**C**) At different time points in CCl4-induced liver fibrosis mice with EGFP-MSCs injected via tail vein, mice were sacrificed and frozen section of the liver tissue was made, and then the homing of EGFP-MSCs were observed under fluorescence microscope (**D**).

As shown in Fig. 4C, fibrotic liver tissue had much higher expression of SDF-1α than normal liver tissue by immunohistochemistry. To determine whether SDF-1α/CXCR4 axis also regulates the recruitment of MSCs in fibrotic liver, we measured the expression level of SDF-1α in liver and BM in CCl4-induced fibrosis model with the time going. We found that as time went on, the expression of SDF-1α was remarkably enhanced in liver, but reduced in BM, and its expressions in the two sites were intersected after the third week (Fig. 5A). Consistent with Real-time PCR results, only when the concentration of SDF-1α in liver was higher than that in BM from the 3rd week, MSCs began to be recruited to fibrotic liver (Fig. 5B,C). Furthermore, AMD3100 administration significantly suppressed MSCs migration to fibrotic liver (Fig. 5D). These data suggested that SDF-1α is the critical cytokine recruiting MSCs from BM to fibrotic liver.

### Endogenic MSCs Also Aggravate the degree of early liver fibrosis in mice

**Figure 6:**
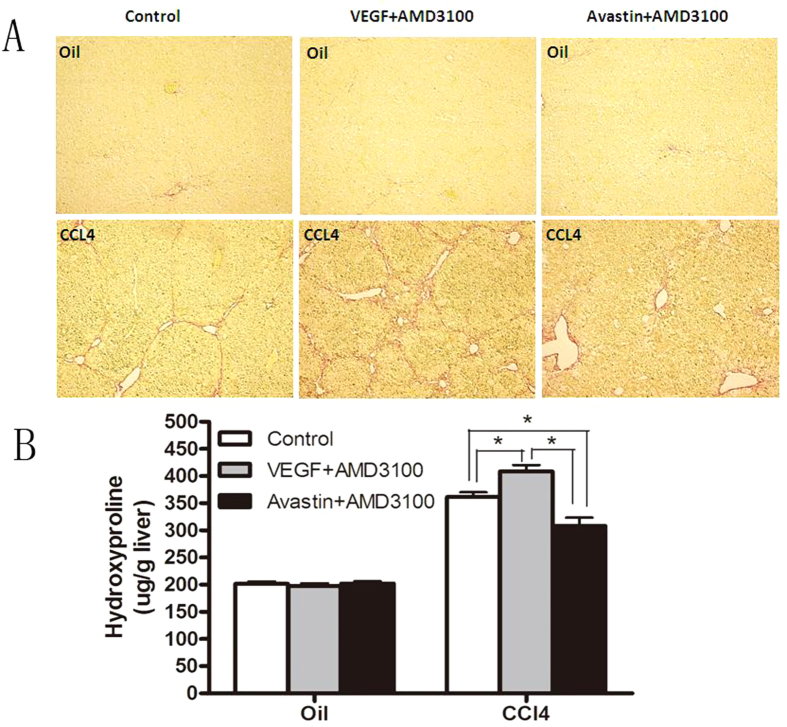
Endogenously induced-MSCs also aggravate liver fibrosis *in vivo*. WT-BALB/c mice were induced hepatic fibrosis by administration of CCl_4_ for 4 weeks. Control group received olive oil treatment. While in the induction of hepatic fibrosis, mobilization of MSCs was carried on. Mice were pretreated with VEGF once daily for 4 days (100 mg/kg i.p.). Twenty-four hours after the last injection, mice were administered AMD3100 (5 mg/kg i.p). After 4 weeks of MSCs mobilization, hepatic fibrosis mice were sacrificed, and the livers were removed to observe the general situation in hepatic cirrhosis. (**A**) The significantly increased amount of ECM was confirmed by Sirius red staining (×100) after MSCs mobilization. Data shown are the representative of 3 animals. (**B**) The amount of liver hydroxyproline was detected in MSCs group compared with that of control groups.

According to previous experiments, we understood that MSCs could proliferate *in vivo* by administrating with VEGF, and then could migrate from BM by transient administrating with CXCR4 antagonist. These methods enable us to artificially induce endogenic MSCs *in vivo*, by which we can address if endogenic MSCs could aggravate the degree of early liver fibrosis in the same way. The results showed that VEGF and AMD3100 could contribute to fibrogenesis, and this effect could be reversed by administrating Avastin or continuous AMD3100 (Fig. 6).

## Discussion

Despite of lots of intense studies, the role of BM-MSCs in liver fibrosis is still a matter of debate. It is reported that MSCs suppress liver fibrosis by differentiation into hepatic cells and by secreting a variety of growth factors and cytokines which can inhibit inflammation, decrease hepatocytes apoptosis, ameliorate fibrosis and improve hepatocytes function^30^,^31^. Meanwhile, there are evidence showing that MSCs can contribute to liver fibrosis by differentiation into myofibroblasts^6^,^32^. We show here that both endogenous and exogenous MSCs could migrate to injured liver and promote liver fibrogenesis in our early liver fibrosis mice model. Although it may finally lead to cirrhosis and hepatic failure, liver fibrosis is actually a wound-healing response following liver injury to repair the tissue and to maintain tissue continuity^33^,^34^. In our study, liver damage was attenuated in MSCs group as indicated by AST and ALT measurement. But it is worth noting that our data only suggested the above mentioned effects in an early liver fibrosis mice model. Whether MSCs play the same role in fibrogenesis and liver damage in advanced cirrhosis warrants further research.

It was reported by Pitchford *et al.* that VEGF, a very important angiogenic factor, may contribute to mobilization of progenitor cell subsets from bone marrow and these cell subsets were not hematopoitic progenitor cells (HPCs)^26^. We speculate that these cell subsets may be MSCs or including MSCs at least. We found that VEGF was highly expressed in livers of CCl4-treated mice and our study provided both *in vitro* and *in vivo* evidence showing that VEGF significantly enhanced MSCs proliferation, which could be attenuated by administration of VEGF monoclonal antibody Avastin, without any effects in MSCs migration.

Chronic liver injury are accompanied by a prominent inflammatory response including an increased expression of CC and CXC chemokines, like CCL21, CXCL9, CXCL16, CXCL12 (SDF-1α) and so on^20^,^35^,^36^,^37^. It is well known that chemokines and their receptors are closely related to hepatic fibrosis. In our study, we screened the key chemokines and receptors contributing to the migration of MSCs from CC and CXC families. The expression of chemokine receptors on MSCs indicated the chemotactic capability to their ligands. Although our results showed that there were many receptors expressed on MSCs and several chemokines were detected in BM and liver, the expression of SDF-1α/CXCR4 is much significantly higher than others. Our study provides compelling evidence for the vital effect of SDF-1α/CXCR4 in MSCs migration. The retention of MSCs within the bone marrow is mainly dependent on the SDF-1α/CXCR4 chemokine axis. Administration of AMD3100, the CXCR4 antagonist, could cause the release of MSCs from BM into the blood. Thus, mechanisms that disrupt this axis might promote the migration of MSCs from the bone marrow niche. As hepatic fibrogenesis is a complex response mediated by many different cell populations, and with the injury aggravating, these different populations are involved in the construction of fibrosis gradually. Hepatic stellate cells (HSCs), which are aboriginal in liver, are one of critical cell populations contributing to fibrogenesis^34^. At the beginning of fibrotic damage, HSCs activation might be the earliest event for fibrogenesis and be earlier than MSCs mobilization. It can explain why fibrosis appeared before MSCs recruitment to liver. Our study showed that migration of MSCs happened by the time that SDF-1α level in liver was higher than that in BM after 3-week CCl4 administration in our model, which means that MSCs could migrate when the balance of SDF-1α was disrupted by a certain degree of liver damage. Therefore, the conclusion might be drawn that SDF-1α is the key cytokine to promote MSCs migration to injured liver.

In conclusion, our data show that MSCs aggravate liver fibrosis and attenuate liver damage in our CCl4-induced liver fibrosis mice model. VEGF is the key cytokine that contributes to MSCs proliferation. SDF-1α/CXCR4 axis plays a key role in regulating MSCs migration from BM to fibrotic liver. These results provide further evidence in the role of MSCs in liver firbosis and elucidate the mechanism underlying MSCs mobilization under the condition of CCl4-induced liver injury.

## Additional Information

**How to cite this article**: Liu, Y. *et al.* Contribution and Mobilization of Mesenchymal Stem Cells in a mouse model of carbon tetrachloride-induced liver fibrosis. *Sci. Rep.* **5**, 17762; doi: 10.1038/srep17762 (2015).

## Supplementary Material

Supplementary Information
