# Supplementary material for: Contribution and Mobilization of Mesenchymal Stem Cells in a mouse model of carbon tetrachloride-induced liver fibrosis
Source: Sci Rep. 2015 Dec 8;5:17762. doi: 10.1038/srep17762 (PMC4672342; doi:10.1038/srep17762)
Supplement: Supplementary Information [file srep17762-s1.pdf]

Contribution and Mobilization of Mesenchymal Stem Cells in a mouse model of carbon tetrachloride-induced liver fibrosis

Yan Liu [1,*], Zhipeng Han[1,*], Yingying Jing [1,*], Xue Yang[1], Shanshan Zhang[1], Chen Zong[1], Jinghua Jiang[1], Kai Sun[2], Rong Li[1], Lu Gao[1], Xue Zhao[2], Dong Wu[3], Yufang Shi[4], Lixin Wei[1]

MSCs Derived From BM are Present in Fibrotic Liver

Next, we investigated whether MSCs are also present in human fibrotic tissues. It is reported that SSEA-4, an early embryonic glycolipid antigen, commonly used as a marker for undifferentiated pluripotent human embryonic stem cells and cleavage to blastocyst stage embryos, is also used to identify the adult mesenchymal stem cell population [20]. In order to verify this marker, we isolated, cultured and purified MSCs from donors' bone marrow and identified them by detecting surface marker CD29+, CD34-, CD44+, CD45-, CD105+, CD90+, CD140a+ (data not shown). These cells were adherent to the plastic surface of the cell culture dish and had a spindle-shaped fibroblastic morphology (Figure S2A upper left). Then we demonstrated that SSEA-4 could mark human MSCs(HMSCs) (Figure S2A) and the ratio of expression was approximately 75% in HMSCs (Figure S2B). This shows that SSEA-4 can identify most of MSCs in human tissue samples. Immunohistochemistry and immunofluorescence results showed that there were SSEA-4 positive cells in the junction of normal and fibrotic liver area (Figure S3A). These cells were sorted with the marker SSEA-4 by flow cytometry from liver fibrosis tissue (Figure S3B). They were identified as MSCs that were capable of differentiating into osteoblasts and adipocytes (Figure S3C). Additionally, these cells expressed CD29, CD90, CD105, and did not express CD34, CD45 (Figure S3D). These data collectively suggest that there are HMSCs in human cirrhotic liver tissues.

VEGF is the Key Cytokine regulating MSCs Proliferation during liver fibrogenesis.

During liver fibrosis or cirrhosis, MSC recruitment is a continuous process. Therefore, MSCs proliferation is required and might be stimulated during this activity. Studies have reported that some angiogenesis related cytokines like vascular endothelial growth factor(VEGF), platelet-derieved growth factor (PDGF) and epidermal growth factor (EGF) could promote MSCs proliferation *in vitro* [21, 22]. VEGF is the key proangiogenesis factor and can regulate other angiogenesis-related cytokines expression. Therefore, we hypothesize that VEGF is one of the key factors promoting MSCs proliferation.

To determine the hypothesis, we analyzed the expression of VEGF in normal and fibrotic liver by immunohistochemical staining. The results demonstrated that VEGF expression in fibrotic tissue was apparently higher than that in normal tissue (Figure S4A). Meanwhile, Real-time PCR and ELISA showed the same trend(Figure S4B). CCK8 detection showed that VEGF could promote

MSCs proliferation compared with control *in vitro* and the effect could be reversed by VEGF monoclonal antibody, Avastin (Figure S4C). To investigate the proliferation-promoting effect of VEGF *in vivo*, we used bone marrow transplantation(BMT) mice model. Mice were administrated with VEGF 100ug/kg four times a week with and without Avastin 200ug/kg twice a week for two weeks. Flow cytometry analysis was employed to detect the content of GFP-cells in BM-derived MSCs. The results showed that VEGF could increase the content of MSCs by 2 fold in BM and the effect could be reversed by Avastin (Figure S4D). Consistently, the proliferation-inhibiting effect of Avastin was also observed in CCl4-induced fibrosis model (Figure S4E). These experiments show that VEGF contributes to MSCs proliferation during liver fibrogenesis.

VEGF Treatment Does Not Affect The Migration of MSCs

We next observed whether VEGF had any effects on MSCs migration. The migratory ability of MSCs in vitro did not alter upon VEGF pretreatment by using wound healing assay and Transwell assay (Figure S5A, 5B). Then we detected the number of GFP-cells in peripheral blood in mice. Compared to BMT group, the number of GFP-cells did not significantly increase in BMT-mice pretreated with VEGF (Figure S5C). These data suggest that VEGF cannot affect the migration of MSCs in vitro and in vivo.

Supplementary figure 1 Identification of mice MSCs

(A) MSCs at passage 8 were grown under different conditions that favor differentiation into either adipocytes (for 14 days), or osteoblasts (for 14 days), as described in Materials and Methods. The presence of triglycerides, characteristic of adipocytes, was revealed by staining with oil red O. Calcium deposition, indicative of osteoblasts, was stained with Von Kossa stain. (B) MSCs derived from BALB/c mice were stained with commercially-available antibodies to analysis the surface marker by flow cytometry (red). Corresponding antibodies of the same isotype were used as controls (white).

Supplementary figure 2 Expression of SSEA-4 in human MSCs

(A) IFC was employed to examine the SSEA-4 expression in human MSCs. (B) Quantification of three independent experiments is shown. (*$P < 0.05$)

Supplementary figure 3 MSCs are also present in human cirrhotic liver.

(A) IHC and IFC were used to detect SSEA-4$^+$ cells in paraffin-embeded hepatic cirrhosis tissues. (B) Flow cytometry was used to analyse the percentage of SSEA-4$^+$ cells in hepatic cirrhosis tissues. SSEA-4$^+$ cells were isolated from fresh hepatic cirrhosis tissues and cultured *in vitro*. (C)These cells were grown under different conditions that favor differentiation into either adipocytes (for 14 days), or osteoblasts (for 14 days), as described in Materials and Methods. The presence of triglycerides, characteristic of adipocytes, was revealed by staining with oil red O. Calcium deposition, indicative of osteoblasts, was stained with Von Kossa stain. (D) Flow

cytometry analysis found that majority of these cells expressed CD90, CD105, CD29, and were negative for CD34, CD45.

Supplementary figure 4 VEGF secreted from injured liver contributes to MSCs proliferation in BM.

(A-B)With the development of hepatic fibrosis, Real-time PCR and IHC were employed to detect the VEGF expression at the 0th, 2nd, 4th, 6th and 8th week. Serum concentration of VEGF was examined with ELISA. (C)Trypan blue staining demonstrated that compared with control group, VEGF could remarkably increase MSCs viability, while Avastin, which is VEGF-specific neutralizing antibody, could inhibit the promotive effect of VEGF on the growth of MSCs. (D) WT-BALB/c mice were lethally irradiated and received BMT including EGFP-positive BMSCs and EGFP-negative whole BM cells. Flow cytometry analysis was used to detect the percentage of EGFP-positive BMSCs in bone marrow after VEGF administration with or without Avastin treatment. (E) Hepatic fibrosis was induced by administration of CCl_4 for 4 weeks. Then flow cytometry was used to detect the percentage of EGFP-positive BMSCs in bone marrow with or without Avastin treatment. BMT, bone marrow transplantation. (*P <0.05, **P <0.01)

Supplementary figure 5 VEGF does not affect MSCs migration from BM.

(A) The wound healing assay was employed to determine the migration of MSCs. MSCs were monitored at the 0th, 48th and 72th hour, which were co-cultured with or without VEGF to determine the rate of migration into the scratched area. (B) The effect of VEGF on invasiveness of MSCs was determined using Transwell assay. A representative photograph is shown in the left panel. Quantification of three independent experiments is shown in the right panel. (×200 magnification). (C) Flow cytometry analysis was used to detect the percentage of EGFP-positive BMSCs in peripheral blood in BMT mice with or without VEGF administration.

Fig. s1

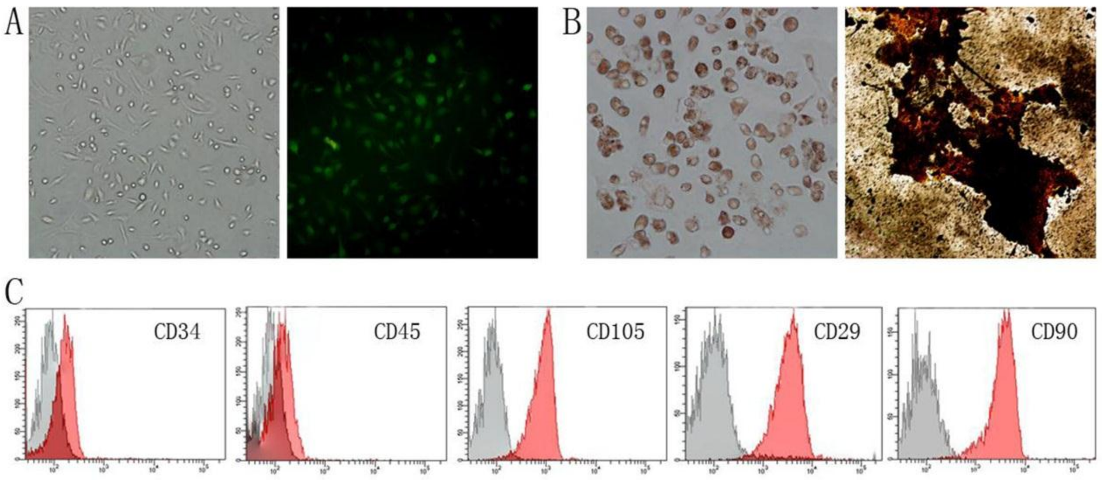

Fig. s2

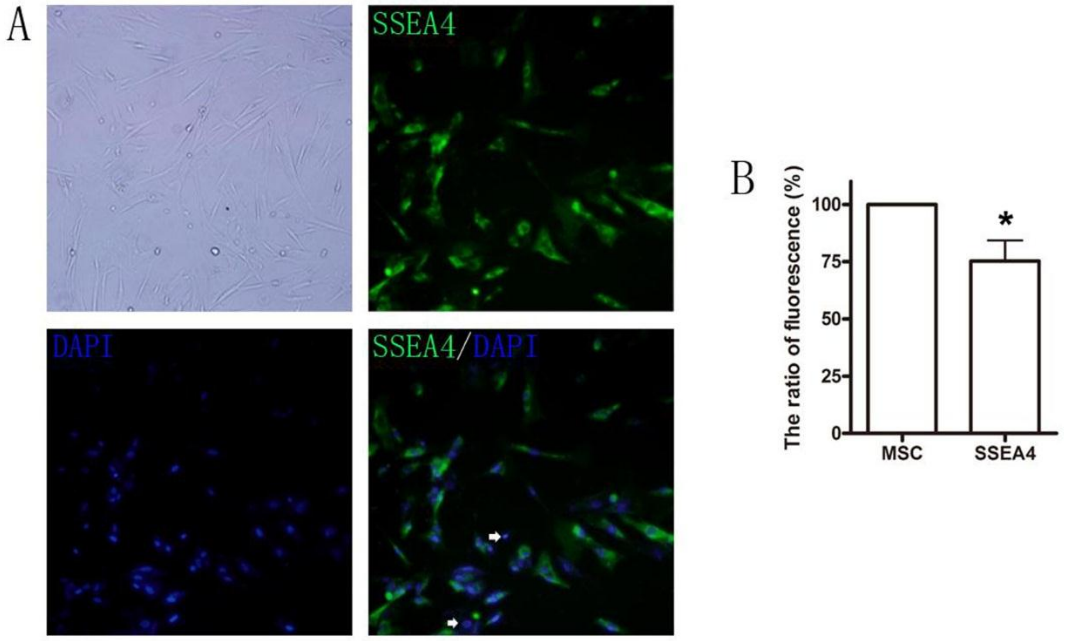
A
SSEA4
DAPI
SSEA4/DAPI
B
The ratio of fluorescence (%)
100
75
50
25
0
*
MSC
SSEA4

Fig. s3

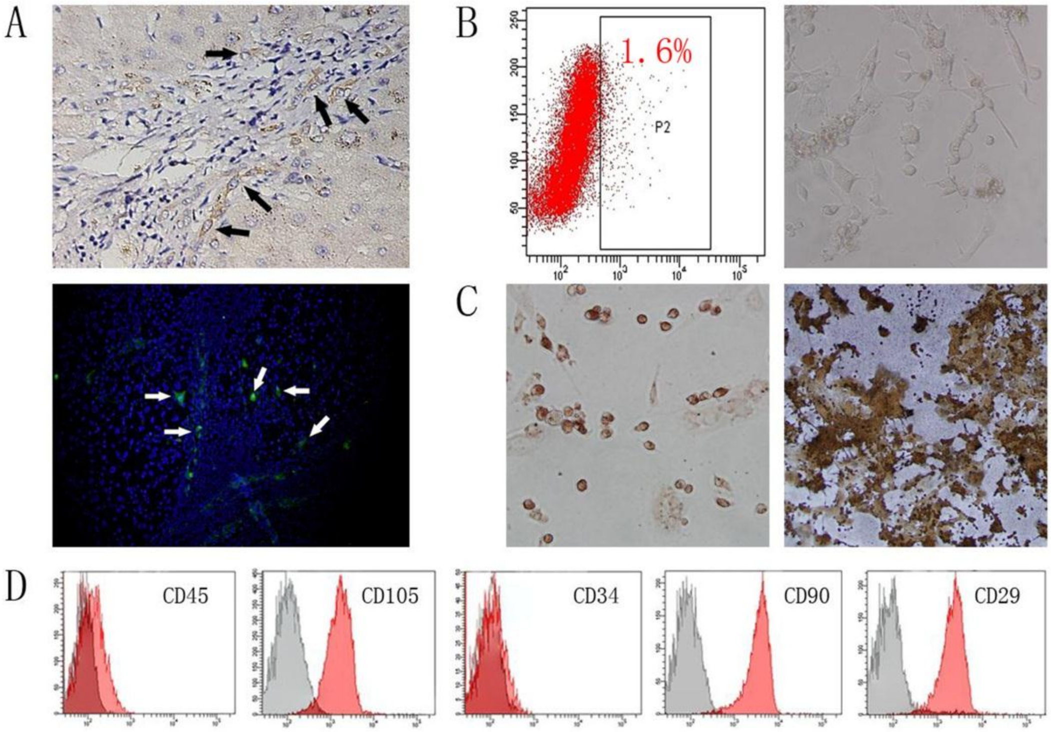
A
B
1.6%
P2
C
D
CD45
CD105
CD34
CD90
CD29

Fig. s4

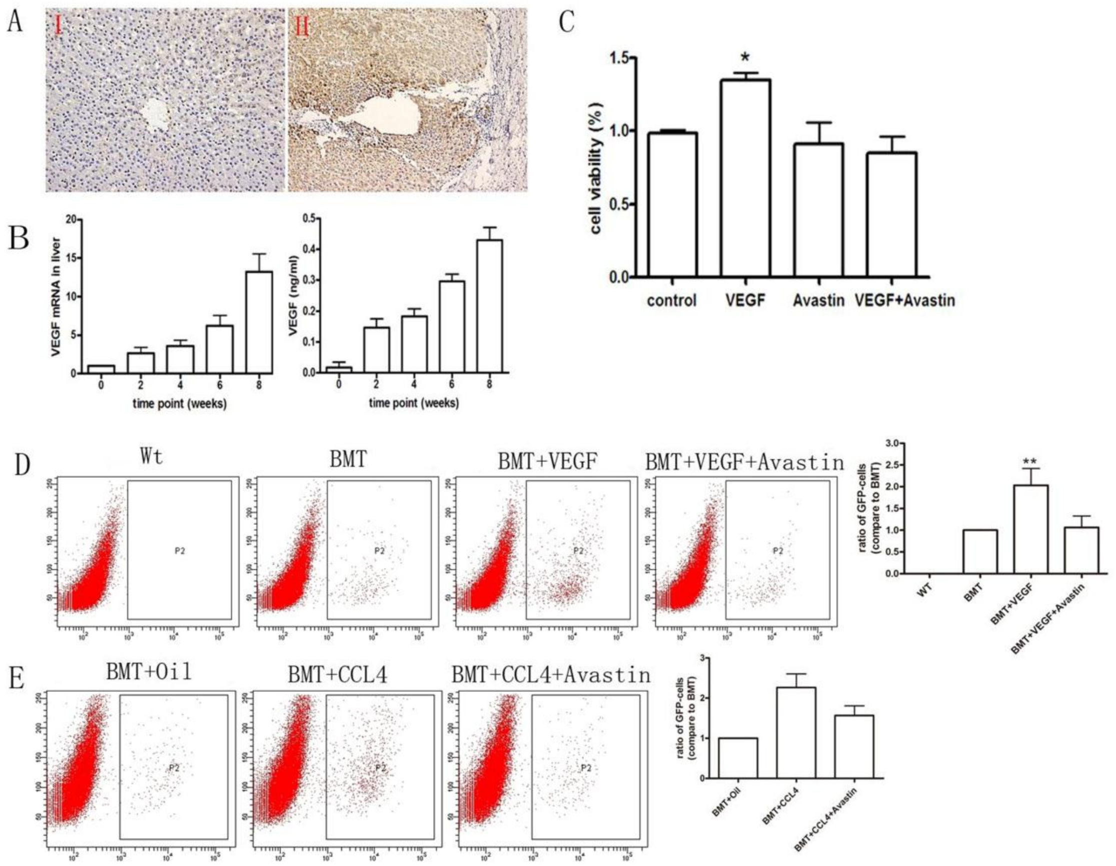
A
I
II
B
VEGF mRNA in liver
time point (weeks)
VEGF (ng/ml)
time point (weeks)
C
cell viability (%)
control
VEGF
Avastin
VEGF+Avastin
D
Wt
BMT
BMT+VEGF
BMT+VEGF+Avastin
P2
ratio of GFP-cells (compare to BMT)
WT
BMT
BMT+VEGF
BMT+VEGF+Avastin
E
BMT+Oil
BMT+CCL4
BMT+CCL4+Avastin
ratio of GFP-cells (compare to BMT)
BMT+Oil
BMT+CCL4
BMT+CCL4+Avastin

Fig. s5

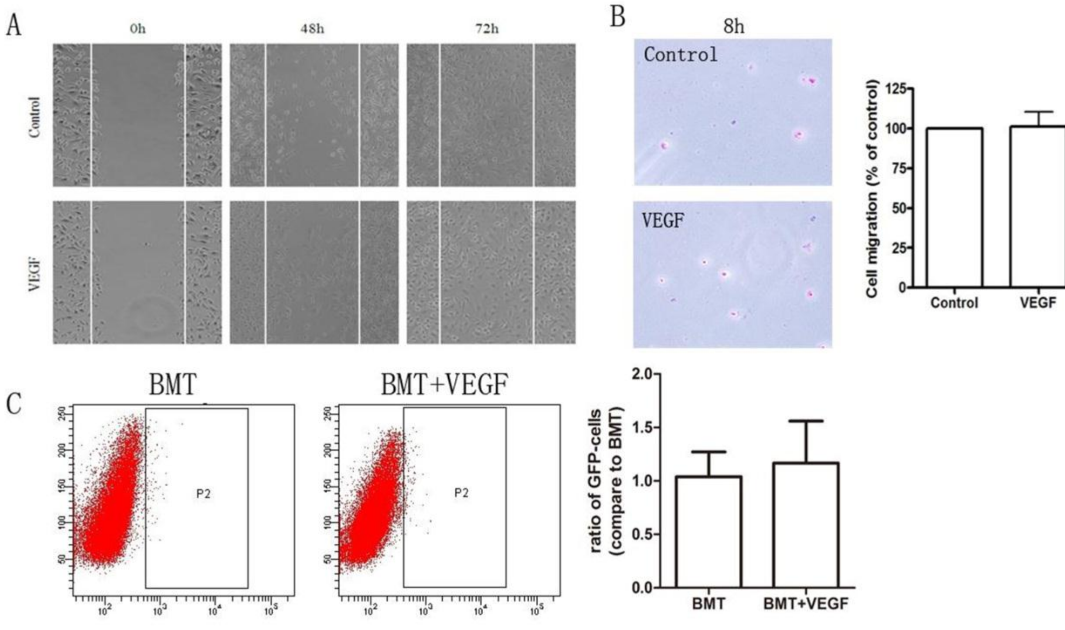
A
0h
48h
72h
Control
VEGF
B
8h
Control
VEGF
Cell migration (% of control)
Control
VEGF
C
BMT
BMT+VEGF
P2
ratio of GFP-cells (compare to BMT)
BMT
BMT+VEGF

Supplementary Table 1. Oligonucleotide sequences of primers of chemokine receptors used in Real-time PCR

Genes	Sequences (forward)	Sequences (reverse)
Ccr1	GACCAGCATCTACCTGTTCA	GCAGAAACAAATACACTCAG
Ccr 2	GTTACCTCAGTTCATCCA	CAAGGCTCACCATCATCGTAGTC
Ccr 3	TTGCAGGACTGGCAGCATT	TTCATTCTTAGAGCATGGAAACGTT
Ccr 4	TCATGACTTCCGTGACGCTTT	GTTTTCTTCCTCAGAGCCCTGTT
Ccr 5	GACATCGATTATGGTATG	GAAGAGCAGGTCAGAGATGGC
Ccr 6	TCGTCCAGGCAACCAAATCTTTCC	TGTTGTATGCGTTTATTGGCCAGA
Ccr 7	CACGCTGAGATGCTCACTGG	CCATCTGGGCCACTTGGA
Ccr 8	TGACCGACTACTACCCTGATTTCTT	GCTGCCCCTGAGGAGGAA
Ccr 9	GTCAGCTGTCTTGATCCTGAAG	CATAGAGAACTGGGTTCAGACAA
Ccr 10	AGAGCTCTGTTACAAGGCTGATGTC	CAGGTGGTACTTCCTAGATTCCAGC
Cxcr1	CTGCTATGAAGTCCTGGGTG	TCAATCAAGTGGGCTCCTAA
Cxcr 2	GTGCCGCTGCTCATCATG	AAGGACGACAGCGAAGATGAC
Cxcr 3	TCCCAACCACAAGTGCCAAAG	AGAAAGGCAAAGTCCGAGGC
Cxcr 4	GGCTGTAGAGCGAGTGTTGC	GTAGAGGTTGACAGTGTAGAT
Cxcr 5	AAACGAAGCGGAAACTAGAGCC	GCCCAGCTTGGTCAGAAGCC
Cxcr 6	TACGATGGGCACTACGAGGGAG	GCAAAGAAACCAACAGGGAGAC

Supplementary Table 2. Oligonucleotide sequences of primers of chemokines used in Real-time PCR

ligand		Sequence	receptor
CCL3/	Sence	CAGCCTTTGCTCCCAGCCAGG	CCR1
MIP1 α	Antisence	AGCCCCTGCTCTACACGGGAC	
CCL4/	Sence	GCTCTGCGTGTCTGCCCTCT	
MIP1 β	Antisence	CTGTGAAGCTGCCGGGAGGT	
CCL2	Sence	CGGCTGGAGCATCCACGTGT	CCR2
	Antisence	CTTTGGGACACCTGCTGCTGGT	
CCL5/	Sence	TGCCTACCTCTCCCTCGCGC	CCR5
RANTES	Antisence	GGCACACACTTGGCGGTTCCT	
CCL21	Sence	CGGCTGCAGGAAGAACCGGG	CCR7
	Antisence	ATCTCCTGGGCTCCAGGCGG	
CXCL12/	Sence	ACAGACAAGTGTGCATTGACCCGA	CXCR4
SDF-1 α	Antisence	ATCGGCAGGAAGCGGGGAACT	
CXCL16	Sence	TGAGCTTGGCACGGATCAGCGC	CXCR6
	Antisence	CAACTTCCAGCGACACTGCCCT	
